# Relationship between body mass index and depressive symptoms: the “fat and jolly” hypothesis for the middle-aged and elderly in China

**DOI:** 10.1186/s12889-016-3864-5

**Published:** 2016-11-29

**Authors:** Lin Zhang, Kun Liu, Hong Li, Dan Li, Zhuo Chen, Li-li Zhang, Lei-lei Guo

**Affiliations:** 1 Department of Community Nursing, School of Nursing, Jinzhou Medical University, No.40, Section 3, Songpo Road, Linghe District, Jinzhou City, Liaoning Province People’s Republic of China; 2 Department of Surgical Nursing, School of Nursing, Jinzhou Medical University, No.40, Section 3, Songpo Road, Linghe District, Jinzhou City, Liaoning Province People’s Republic of China; 3 Department of Experimental Center, School of Nursing, Jinzhou Medical University, No.40, Section 3, Songpo Road, Linghe District, Jinzhou City, Liaoning Province People’s Republic of China; 4 Department of Surgery, Third Affiliated Hospital of Jinzhou Medical University, No.28, Section 2, Chongqing Road, Linghe District, Jinzhou City, Liaoning Province People’s Republic of China

**Keywords:** Body mass index, Depressive symptoms, Fat and jolly, Middle-aged and elderly, Obesity

## Abstract

**Background:**

Obesity has been identified as a worldwide epidemic. In China, the highest prevalence of obesity is observed in adults aged ≥45 years old. This study aimed to describe the association between BMI and depressive symptoms among a large representative sample of middle-aged and elderly in China.

**Method:**

A longitudinal sample of the middle-aged and elderly (6,224 males and 6,883 females) who were interviewed in the 2011 China Health and Retirement Longitudinal Study was used. A multivariate logistic regression analysis was used to examine the effects of socio-demographic characteristics, lifestyle, activity status, health status, physical exercise and body weight on depressive symptoms.

**Results:**

Approximately 6.94% of the males were underweight, 25.48% were overweight and 8.16% were obese. A higher prevalence of obesity was found among women, with 6.89% being underweight, 31.98% overweight and 14.28% obese. The underweight subjects were more likely to be depressed (odds ratio; OR = 1.30 and 1.19) compared with the normal weight people, respectively, whereas overweight and obese men and women were less likely to be depressed (overweight: OR = 0.76 and 0.80; obesity: OR = 0.64 and 0.65, respectively) than people of normal weight.

**Conclusion:**

Our data are consistent with the “fat and jolly” hypothesis being valid in both middle-aged and elderly men and women.

## Background

Recently, being overweight and obese has become an important public health concern in many countries. In China, the proportion of overweight and obese adults is 21.8% [1]. Overweight and obese increase the risk of heart disease, strokes, diabetes and liver disease and individuals are far more likely to experience discrimination than health peers [2]. This discrimination known as negative attitudes, beliefs and behaviors towards individuals with obesity is widespread [3]. Weight discrimination occurs in a range of settings, including medical, educational, and interpersonal contexts, and it is associated with a range of adverse personal, social, and economic outcomes [4, 5].

The Chinese population has begun aging and will continue to age rapidly in the future. Population aging creates the challenges of economic support and elderly care. However, healthy aging can potentially lessen both of these burdens [6]. More efforts and attempts are needed to change “weight bias” and prompt healthy aging. According to Chinese chronological tradition, there is a saying: “laughing and growing fat is a blessing”. Compared to Western culture, being slightly overweight in China has been regarded as healthy because only wealthy people can afford to gain weight. No evidence exists that supports the relationship between fat and jolly.

Conventional wisdom suggests that obesity has become a major contributor to a variety of health problems such as depression. Major depressive symptoms increases the risk of mortality [7–9], and depressive symptoms studies account for the majority of etiological studies of obesity and psychological morbidity [10]. However, the relationship between obesity and depressive symptoms is controversial, with several studies showing positive, null or negative associations. The “fat and jolly” hypothesis [11] proposes that obesity has a negative association with depressive symptoms and results in a reduction in depressive symptoms. Dong Q [12] conducted a study to investigate the correlation between obesity and depressive symptoms in a representative sample of China’s elderly population (aged ≥60 years) and found that their study supported the “fat and jolly” hypothesis only in rural older Chinese men but not in women. Using a cross-sectional dataset of 2,604 people aged ≥55 years in China, Ho [13] found that both obese men and women were less likely to suffer from depressive symptoms compared to normal-weight individuals. Another research study conducted by Yu [14]showed that their findings supported the “fat and jolly” hypothesis in both genders among adults (aged 18-64 years) in the Taiwanese community. Similar evidence has been found in Hong Kong and other Taiwanese studies. Based on an elderly sample population, Li [15] and Chang [16] both found that obese men and women were less likely to suffer from depressive symptoms than normal weight individuals. However, contradictory evidence has been found in other Asian countries. Based on a sample of adults aged 60-85 in Korea, Kim [17] found a negative association between body mass index (BMI) and depressive symptoms in women but not in men. A “U-shaped” association between BMI and depressive symptoms has also been found by Noh [18] among women and men aged 50-102 years old in Korea. However, additional studies [19–23] have been inconsistent. Several studies have also addressed this topic in Western countries such as the UK [11, 24], France [25], the United States [26–38], Canada [39], Finland [40], New Zealand [41], Germany [42], Australia [43], and Poland [44]. These studies have reported mixed results regarding the association between body weight and depressive symptoms. For example, in a sample of men aged 40 to 59, Crisp [11] found that the obese were less depressed than the non-obese. Revah-Levy [25], using data from a representative sample of 39,542 adolescents aged 17, found a “U-shaped” association between BMI and depressive symptoms in boys but a negative association in girls. Palinkas [29] found no association between obesity and depressive symptoms in women but found a negative association in men. However, using data from the 1992National Longitudinal Alcohol Epidemiologic Study, Carpenter [30] found that being overweight increased depressive symptoms in women but decreased depressive symptoms in men. Jorm [43] found no association between obesity and depressive symptoms in females aged 20-64 in Australia but found a positive association in males. Luppino FS [45] conducted a systematic review and meta-analysis on the longitudinal relationship between depressive symptoms, being overweight, and obesity, and this meta-analysis confirmed a reciprocal link between depressive symptoms and obesity. Obesity was found to increase the risk of depressive symptoms. In addition, depressive symptoms was found to be predictive of developing obesity.de Wit L [46] conducted a meta-analysis of cross-sectional studies in the general population to examine the nature of the association between depressive symptoms and obesity, and they found a significant positive association for females and a smaller non-significant association for males. A significant positive association was found between depressive symptoms and obesity in the general population, which appeared to be more marked among women.

No consistent recognition of the association between BMI and depressive symptoms exists. Therefore, this article focused on the relationship between depressive symptoms and obesity among the middle-aged and elderly in China after adjusting for potential confounders. Furthermore, this work explores the stability of the association between BMI and depressive symptoms by gender.

## Methods

### Study participants

The China Health and Retirement Longitudinal Study (CHARLS) is a nationally representative longitudinal survey of a middle-aged and elderly population and their spouses in China. The respondents are followed-up every 2 years using a face-to-face, computer-aided personal interview (CAPI). The CHARLS baseline survey had a four-stage, stratified, cluster probability sampling design. In the first stage, all of the counties in China were stratified by region, rural/urban status and per capita statistics on gross domestic product. A random sample of 150 counties was selected to represent the socio-economic and geographic pattern of all of the counties. In the second stage, three PSUs were selected in each county with the probability proportional to their population size. In the third stage, all of the households in each selected PSU were mapped, and a random sample of 24 households was selected among all of the households with residents aged ≥45 years within each PSU. Finally, for each selected household, one resident aged ≥45 years was randomly selected as a participant in the survey [47]. The 2011 Wave1of China Health and Retirement Longitudinal Study (2011 CHARLS Wave1) was conducted in 2011.Fromthe 2011 CHARLSWave1. We included a total of 13,107 individuals in our study.

### Anthropometric measures

Weight and height were measured using a weight and height measurement instrument. BMI was calculated as weight in kilograms divided by height in meters squared. Using the standard Chinese definition, BMI was categorized into four groups: underweight (BMI < 18.5), normal (18.5 ≤ BMI < 25), overweight (25 ≤ BMI < 30), and obese (BMI > 30) [48].

### Socio-demographic and occupational factors

A range of adverse personal, social, and economic outcomes has shown to be a confounder of the association between BMI and depressive symptoms [10].

Data including age, gender, education, marital status, hukou, current residence, smoking habit, alcohol consumption, average hours of sleep per night, eating habits, activity status, major accidental injury, chronic diseases, health status, and regular physical exercise were collected by the self-reported questionnaire. (a)The participants were grouped according to the following categories: age was categorized as younger than 45, 45-49, 50-54, 55-59, 60-64, 65-69, and 70 and older. (b) Educational level was classified into four groups: illiterate, less than elementary school, high school, and above vocational school. (c) Marital status was divided into two groups: married and single (never married, divorced, widowed or separated). (d) Hukou is an important part of household registration in our country and is strictly enforced. Non-agricultural hukou people primarily live in the city and work in factories, public institutions and other workplaces. They have no farm land but have the convenience of city life. Agricultural hukou people primarily live in villages and depend on agriculture and farming for their livelihood. Hukou was categorized into two groups: non-agricultural hukou and agricultural hukou. (e) Current residence was classified as rural or urban. (f) Smoking habit was divided into current smoker, ex-smoker and never smoked. (g) Alcohol consumption was grouped as more than once a month, less than once a month, and non-drinker. (h) Average hours of sleep per night was defined as the mean actual sleep during the past month. (i) Eating habits were categorized into three groups: 2 meals per day or less, 3 meals per day, and 4 meals per day or more. (j) Activity status (including performing voluntary or charity work, caring for a sick or disabled adult, providing help to family, friends or neighbors, attending an educational or training course, interacting with friends, going to a sporting event, social or other type of club, and participating in a community-related organization) was dichotomized (at least once a month) versus never. (k) Major accident information was obtained by asking the participant whether he/she suffered from any type of major accidental injury and received medical treatment, the answer was “yes” or “no”. (l) Following Chang (2012) [16], a continuous variable was used that reflects the presence of chronic health conditions by assessing 14 common chronic symptoms among the middle-aged and elderly, including hypertension, dyslipidemia, diabetes or high blood sugar, cancer or malignant tumor, chronic lung diseases, liver disease, heart problems, stroke, kidney disease, digestive disease, emotional, nervous, or psychiatric problems, memory-related disease, arthritis or rheumatism, and asthma. The presence of each symptom is coded as one, and the sum of scores for all symptoms, which ranges from 0 to 14, was used as an indicator of disease. (m) Self-reported health status was divided into five groups: very good, good, fair, poor, and very poor. (n) Regular physical exercise was defined as exercising at least 3 days per week and more than 30 min per day, including moderate to vigorous physical activity and walking.

### Depressive symptoms measures

The Chinese version of the Center for Epidemiologic Studies-Depression scale (CES-D) has been widely used to measure depressive symptoms in China and is highly reliable and valid [6, 49–51]. The CES-D is a self-reported questionnaire that consists of 10 questions. Survey participants with a score of 10 or higher on the CES-D were classified as depressive symptoms [52]. The Cronbach alpha coefficient was 0.86 and the construct validity was 0.62.

### Statistical analysis

Age, average hours of sleep per night, diseases (0-14), BMI, and CES-D scores were expressed as the mean and standard deviation. To evaluate the relationship between related factors and depressive symptoms, Data were analyzed using chi-square test analyses of variance followed by Bonferroni adjustment.

A multivariate logistic regression analysis was used, with the BMI groups as the independent variable and depressive symptoms as the dependent variable, to evaluate the relationship between obesity and depressive symptoms. For the multivariate logistic regression analysis, the BMI groups were compared with the normal weight group as a reference. To evaluate the probable interactions between BMI, sex groups, and depressive symptoms (7, 17), a general linear model was used. We found an interaction between BMI and sex groups (*P* = 0.000). Therefore, we analyzed our data separately according to gender in the multivariate logistic regression analysis. After adjusting for age, education, marital status, hukou, current residence, smoking habit, alcohol consumption, average hours of sleep per night, eating habits, activity status, major accidental injury, chronic diseases, self-reported health status, and regular physical exercise, the odds ratios (ORs) were calculated to investigate the adjusted relationship between obesity and depressive symptoms.

## Results

### Socio-demographic and baseline characteristics of the subjects

In total, 13,107 participants who effectively completed the questionnaires were included in our study. The distribution of demographic variables is shown in Table 1. Overall, 6,224(47.49%) of the participants were male, and 6,883 (52.51%) of the participants were female. The average ages of the male and female participants were 59.91 ± 9.38 and 58.95 ± 9.67 years, respectively. The mean value of the CES-D scores was 10.01 ± 4.86. Among all of the participants, 6.91% were underweight, 28.89% were overweight and 11.38% were obese. Furthermore, regarding the males, 6.94% were underweight, 25.48% were overweight and 8.16% were obese, whereas 6.89%, 31.98% and 14.28% of the females were underweight, overweight, and obese, respectively. Significant differences in distribution were observed between males and females in all of the variables, except eating habits, activity status, and regular physical exercise. Tables 2 and 3 show the socio-demographic and baseline characteristics of the participants. Only having experienced a major accidental injury and partaking in regular physical exercise were not significantly different between the BMI groups in males (Table 2), and in females, only having experienced a major accidental injury was not significantly different between the BMI groups (Table 3). The prevalence of depressive symptoms, assessed by the CES-D, differed among the BMI groups. Table 4 shows the significantly different variables among the BMI groups.

**Table 1:** Baseline characteristics with full samples

Variables	Male	Female	Total	*t/*χ^2^	*P*
	N (%)	N (%)	N (%)		
N	6224 (47.49)	6883 (52.51)	13107 (100)		
Age (years)	59.91 ± 9.38	58.95 ± 9.67	59.40 ± 9.55	5.785	0.000
Average hours for one night	6.47 ± 1.81	6.24 ± 1.95	6.35 ± 1.89	6.982	0.000
Diseases (0-14)	1.30 ± 1.34	1.46 ± 1.41	1.38 ± 1.38	-6.241	0.000
CESD	7.48 ± 5.84	9.47 ± 6.56	8.53 ± 6.31	-18.318	0.000
BMI	22.97 ± 3.70	23.96 ± 4.16	23.49 ± 3.98	-14.341	0.000
Age (years)					
45-49	1063 (17.08)	1481 (21.52)	2544 (19.41)	51.737	0.000
50-54	903 (14.51)	1022 (14.85)	1925 (14.69)		
55-59	1297 (20.84)	1422 (20.66)	2719 (20.74)		
60-64	1120 (17.99)	1173 (17.04)	2293 (17.49)		
65-69	782 (12.56)	731 (10.62)	1513 (11.54)		
≥ 70	1059 (17.01)	1054 (15.31)	2113 (16.12)		
Education					
Illiterate	786 (12.63)	2881 (41.86)	3667 (27.98)	1416.286	0.000
Less than elementary school	4537 (72.90)	3488 (50.68)	8025 (61.23)		
High school	560 (9.00)	354 (5.14)	914 (6.97)		
Above vocational school	341 (5.48)	160 (2.32)	501 (3.82)		
Marital status					
Single	604 (9.70)	1103 (16.02)	1707 (13.02)	115.274	0.000
Married	5620 (90.30)	5780 (83.98)	11400 (86.98)		
Hukou					
Non-agricultural hukou	1302 (20.92)	1225 (17.80)	2527 (19.28)	20.464	0.000
Agricultural hukou	4922 (79.08)	5658 (82.20)	10580 (80.72)		
Current residence					
Rural	3999 (64.25)	4264 (61.95)	8263 (63.04)	7.431	0.006
Urban	2225 (35.75)	2619 (38.05)	4844 (36.96)		
Smoke					
NO	1566 (25.16)	6290 (91.38)	7856 (59.94)	5972.096	0.000
Former smoke	1032 (16.58)	159 (2.31)	1191 (9.09)		
Current smoke	3626 (58.26)	434 (6.31)	4060 (30.98)		
Drinking					
NO	2751 (44.20)	6050 (87.90)	8801 (67.15)	2949.096	0.000
Less than once a month	686 (11.02)	355 (5.16)	1041 (7.94)		
More than once a month	2787 (44.78)	478 (6.94)	3265 (24.91)		
Eating meals					
≤ 2 meals per day	813 (13.06)	932 (13.54)	1745 (13.31)	0.722	0.697
3 meals per day	5310 (85.31)	5844 (84.90)	11154 (85.10)		
≥ 4 meals per day	101 (1.62)	107 (1.55)	208 (1.59)		
Taking activities					
No	3057 (49.12)	3458 (50.24)	6515 (49.71)	1.650	0.199
Yes	3167 (50.88)	3425 (49.76)	6592 (50.29)		
Ever been in major accidental injury					
NO	5416 (87.02)	6412 (93.16)	11828 (90.24)	139.885	0.000
Yes	808 (12.98)	471 (6.84)	1279 (9.76)		
Self-report health status					
Very poor	884 (14.20)	1272 (18.48)	2156 (16.45)	92.455	0.000
Poor	2186 (35.12)	2616 (38.01)	4802 (36.64)		
Fair	2092 (33.61)	2100 (30.51)	4192 (31.98)		
Good	824 (13.24)	722 (10.49)	1546 (11.80)		
Very good	238 (3.82)	173 (2.51)	411 (3.14)		
Having regular physical exercises					
No physical exercise	3906 (62.76)	4230 (61.46)	8136 (62.07)	2.391	0.303
Less than regular physical exercises	1145 (18.40)	1318 (19.15)	2463 (18.79)		
Regular physical exercises	1173 (18.85)	1335 (19.40)	2508 (19.13)		
BMI					
Underweight	432 (6.94)	474 (6.89)	906 (6.91)	252.969	0.000
Average	3698 (59.42)	3225 (46.85)	6923 (52.82)		
Overweight	1586 (25.48)	2201 (31.98)	3787 (28.89)		
Obese	508 (8.16)	983 (14.28)	1491 (11.38)		
CESD				256.776	
CES-D < 10	4313 (69.30)	3834 (55.70)	8147 (62.16)		0.000
CES-D ≥ 10	1911 (30.70)	3049 (44.30)	4960 (37.84)		

CESD: Center for Epidemiologic Studies- depressive symptoms scale

*BMI* body mass index, BMI Categories include Underweight (–18.5 kg/m^2^)/Normal weight (18.5–24 kg/m^2^)/Overweight (24–28 kg/m^2^)/Obesity (28– kg/m^2^)

Hukou: it is an important part of household registration in china and is strictly enforced. Non-agricultural hukou people primarily live in the city and work in factories, public institutions and other workplaces. They have no farm land but have the convenience of city life. Agricultural hukou people primarily live in villages and depend on agriculture and farming for their livelihood

**Table 2:** Baseline characteristics in male study population (*N* = 6224)

Variables	Under-weight (%)	Normal weight (%)	Over-weight (%)	Obese (%)	χ^*2*^	*P*
Age (years)						
45-49	29 (6.71)	566 (15.31)	331 (20.87)	137 (26.97)	282.696	0.000
50-54	35 (8.10)	538 (14.55)	260 (16.39)	70 (13.78)		
55-59	55 (12.73)	804 (21.74)	334 (21.06)	104 (20.47)		
60-64	78 (18.06)	652 (17.63)	296 (18.66)	94 (18.50)		
65-69	66 (15.28)	483 (13.06)	181 (11.41)	52 (10.24)		
≥ 70	169 (39.12)	655 (17.71)	184 (11.60)	51 (10.04)		
Education						
Illiterate	95 (21.99)	521 (14.09)	125 (7.88)	45 (8.86)	139.546	0.000
Less than elementary school	294 (68.06)	2731 (73.85)	1145 (72.19)	367 (72.24)		
High school	27 (6.25)	290 (7.84)	193 (12.17)	50 (9.84)		
Above vocational school	16 (3.70)	156 (4.22)	123 (7.76)	46 (9.06)		
Marital status						
Single	70 (16.20)	409 (11.06)	91 (5.74)	34 (6.69)	62.317	0.000
Married	362 (83.80)	3289 (88.94)	1495 (94.26)	474 (93.31)		
Hukou						
Non-agricultural hukou	58 (86.57)	607 (83.59)	475 (70.05)	162 (68.11)	175.167	0.000
Agricultural hukou	374 (13.43)	3091 (16.41)	1111 (29.95)	346 (31.89)		
Current residence						
Rural	318 (73.61)	2568 (69.44)	861 (54.29)	252 (49.61)	175.857	0.000
Urban	114 (26.39)	1130 (30.56)	725 (45.71)	256 (50.39)		
Smoke						
NO	95 (21.99)	832 (22.50)	475 (29.95)	164 (32.28)	159.328	0.000
Former smoke	65 (15.05)	508 (13.74)	333 (21.00)	126 (24.80)		
Current smoke	272 (62.96)	2358 (63.76)	778 (49.05)	218 (42.91)		
Drinking						
NO	226 (52.31)	1590 (43.00)	708 (44.64)	227 (44.69)	19.050	0.000
Less than once a month	39 (9.03)	410 (11.09)	167 (10.53)	70 (13.78)		
More than once a month	167 (38.66)	1698 (45.92)	711 (44.83)	211 (41.54)		
Eating meals						
≤ 2 meals per day	72 (16.67)	537 (14.52)	157 (9.90)	47 (9.25)	55.712	0.000
3 meals per day	346 (80.09)	3089 (83.53)	1416 (89.28)	459 (90.35)		
≥ 4 meals per day	14 (3.24)	72 (1.95)	13 (0.82)	2 (0.39)		
Taking activities						
No	263 (60.88)	1865 (50.43)	699 (44.07)	230 (45.28)	45.621	0.000
Yes	169 (39.12)	1833 (49.57)	887 (55.93)	278 (54.72)		
Ever been in major accidental injury						
NO	374 (86.57)	3232 (87.40)	1359 (85.69)	451 (88.78)	4.431	0.219
Yes	58 (13.43)	466 (12.60)	227 (14.31)	57 (11.22)		
Self-report health status						
Very poor	92 (21.30)	525 (14.20)	199 (12.55)	68 (13.39)	62.035	0.000
Poor	182 (42.13)	1297 (35.07)	527 (33.23)	180 (35.43)		
Fair	116 (26.85)	1266 (34.23)	549 (34.62)	161 (31.69)		
Good	37 (8.56)	484 (13.09)	229 (14.44)	74 (14.57)		
Very good	5 (1.16)	126 (3.41)	82 (5.17)	25 (4.92)		
Having regular physical exercises						
No physical exercise	271 (62.73)	2340 (63.28)	971 (61.22)	324 (63.78)	6.762	0.343
Less than regular physical exercises	87 (20.14)	681 (18.42)	297 (18.73)	80 (15.75)		
Regular physical exercises	74 (17.13)	677 (18.31)	318 (20.05)	104 (20.47)		
CESD						
CES-D < 10	240 (55.56)	2490 (67.33)	1195 (75.35)	388 (76.38)	84.293	0.000
CES-D ≥ 10	192 (44.44)	1208 (32.67)	391 (24.65)	120 (23.62)		

CESD: Center for Epidemiologic Studies- depressive symptoms scale

*BMI* body mass index, BMI Categories include Underweight (–18.5 kg/m^2^)/Normal weight (18.5–24 kg/m^2^)/Overweight (24–28 kg/m^2^)/Obesity (28– kg/m^2^)

Hukou: it is an important part of household registration in china and is strictly enforced.Non-agricultural hukou people primarily live in the city and work in factories, public institutions and other workplaces. They have no farm land but have the convenience of city life. Agricultural hukou people primarily live in villages and depend on agriculture and farming for their livelihood

**Table 3:** Baseline characteristics in female study population (*N* = 6883)

Variables	Under-weight (%)	Normal weight (%)	Over-weight (%)	Obese (%)	χ^2^	P
Age (years)						
45-49	42 (8.86)	661 (20.50)	542 (24.63)	236 (24.01)	254.403	0.000
50-54	36 (7.59)	448 (13.89)	372 (16.90)	166 (16.89)		
55-59	89 (18.78)	682 (21.15)	435 (19.76)	216 (21.97)		
60-64	71 (14.98)	548 (16.99)	390 (17.72)	164 (16.68)		
65-69	70 (14.77)	349 (10.82)	220 (10.00)	92 (9.36)		
≥ 70	166 (35.02)	537 (16.65)	242 (11.00)	109 (11.09)		
Education						
Illiterate	274 (57.81)	1431 (44.37)	824 (37.44)	352 (35.81)	106.379	0.000
Less than elementary school	190 (40.08)	1577 (48.90)	1172 (53.25)	549 (55.85)		
High school	7 (1.48)	154 (4.78)	135 (6.13)	58 (5.90)		
Above vocational school	3 (0.63)	63 (1.95)	70 (3.18)	24 (2.44)		
Marital status						
Single	128 (27.00)	583 (18.08)	275 (12.49)	117 (11.90)	85.360	0.000
Married	346 (73.00)	2642 (81.92)	1926 (87.51)	866 (88.10)		
Hukou						
Non-agricultural hukou	46 (9.70)	500 (15.50)	457 (20.76)	222 (22.58)	61.443	0.000
Agricultural hukou	428 (90.30)	2725 (84.50)	1744 (79.24)	761 (77.42)		
Current residence						
Rural	364 (76.79)	2111 (65.46)	1269 (57.66)	520 (52.90)	112.514	0.000
Urban	110 (23.21)	1114 (34.54)	932 (42.34)	463 (47.10)		
Smoke						
NO	396 (83.54)	2945 (91.32)	2034 (92.41)	915 (93.08)	53.824	0.000
Former smoke	13 (2.74)	72 (2.23)	52 (2.36)	22 (2.24)		
Current smoke	65 (13.71)	208 (6.45)	115 (5.22)	46 (4.68)		
Drinking						
NO	412 (86.92)	2803 (86.91)	1941 (88.19)	894 (90.95)	15.206	0.019
Less than once a month	22 (4.64)	174 (5.40)	120 (5.45)	39 (3.97)		
More than once a month	40 (8.44)	248 (7.69)	140 (6.36)	50 (5.09)		
Eating meals						
≤ 2 meals per day	99 (20.89)	469 (14.54)	257 (11.68)	107 (10.89)	45.651	0.000
3 meals per day	369 (77.85)	2694 (83.53)	1912 (86.87)	869 (88.40)		
≥ 4 meals per day	6 (1.27)	62 (1.92)	32 (1.45)	7 (0.71)		
Taking activities						
No	280 (59.07)	1722 (53.40)	1037 (47.11)	419 (42.62)	59.035	0.000
Yes	194 (40.93)	1503 (46.60)	1164 (52.89)	564 (57.38)		
Ever been in major accidental injury						
NO	439 (92.62)	2993 (92.81)	2056 (93.41)	924 (94.00)	2.155	0.541
Yes	35 (7.38)	232 (7.19)	145 (6.59)	59 (6.00)		
Self-report health status						
Very poor	117 (24.68)	605 (18.76)	376 (17.08)	174 (17.70)	37.831	0.000
Poor	208 (43.88)	1197 (37.12)	831 (37.76)	380 (38.66)		
Fair	103 (21.73)	996 (30.88)	696 (31.62)	305 (31.03)		
Good	36 (7.59)	337 (10.45)	245 (11.13)	104 (10.58)		
Very good	10 (2.11)	90 (2.79)	53 (2.41)	20 (2.03)		
Having regular physical exercises						
No physical exercise	326 (68.78)	1931 (59.88)	1350 (61.34)	623 (63.38)	26.666	0.000
Less than regular physical exercises	82 (17.30)	676 (20.96)	390 (17.72)	170 (17.29)		
Regular physical exercises	66 (13.92)	618 (19.16)	461 (20.95)	190 (19.33)		
CESD						
CES-D < 10	202 (42.62)	1717 (53.24)	1299 (59.02)	616 (62.67)	69.944	0.000
CES-D ≥ 10	272 (57.38)	1508 (46.76)	902 (40.98)	367 (37.33)		

CESD: Center for Epidemiologic Studies- depressive symptoms scale

*BMI* body mass index, BMI Categories include Underweight (–18.5 kg/m^2^)/Normal weight (18.5–24 kg/m^2^)/Overweight (24–28 kg/m^2^)/Obesity (28– kg/m^2^)

Hukou: it is an important part of household registration in china and is strictly enforced.Non-agricultural hukou people primarily live in the city and work in factories, public institutions and other workplaces. They have no farm land but have the convenience of city life. Agricultural hukou people primarily live in villages and depend on agriculture and farming for their livelihood

**Table 4:** Relationship of various characteristics and depressive symptoms, chi-square test analyses of variance followed by Bonferroni adjustment (*N* = 13107)

Variables	CES-D < 10	CES-D ≥ 10	χ^2^	*P*
	N (%)	N (%)		
Gender				
Male	4313 (69.30)	1911 (30.70)	256.776	0.000
Female	3834 (55.70)	3049 (44.30)		
Age (years)				
45-49	1741 (68.44)	803 (31.56)	88.867	0.000
50-54	1251 (64.99)	674 (35.01)		
55-59	1705 (62.71)	1014 (37.29)		
60-64	1358 (59.22)	935 (40.78)		
65-69	880 (58.16)	633 (41.84)		
≥ 70	1212 (57.36)	901 (42.64)		
Education				
Illiterate	1922 (52.41)	1745 (47.59)	366.146	0.000
Less than elementary school	5082 (63.33)	2943 (36.67)		
High school	719 (78.67)	195 (21.33)		
Above vocational school	424 (84.63)	77 (15.37)		
Marital status				
Single	835 (48.92)	872 (51.08)	146.294	0.000
Married	7312 (64.14)	4088 (35.86)		
Hukou				
Non-agricultural hukou	6268 (59.24)	4312 (40.76)	198.071	0.000
Agricultural hukou	1879 (74.36)	648 (25.64)		
Current residence				
Rural	4793 (58.01)	3470 (41.99)	163.867	0.000
Urban	3354 (69.24)	1490 (30.76)		
Smoke				
NO	4657 (59.28)	3199 (40.72)	69.383	0.000
Former smoke	800 (67.17)	391 (32.83)		
Current smoke	2690 (66.26)	1370 (33.74)		
Drinking				
NO	5201 (59.10)	3600 (40.90)	108.573	0.000
Less than once a month	694 (66.67)	347 (33.33)		
More than once a month	2252 (68.97)	1013 (31.03)		
Eating meals				
≤ 2 meals per day	878 (50.32)	867 (49.68)	121.020	0.000
3 meals per day	7129 (63.91)	4025 (36.09)		
≥ 4 meals per day	140 (67.31)	68 (32.69)		
Taking activities				
No	3783 (58.07)	2732 (41.93)	92.198	0.000
Yes	4364 (66.20)	2228 (33.80)		
Ever been in major accidental injury				
NO	7437 (62.88)	4391 (37.12)	26.610	0.000
Yes	710 (55.51)	569 (44.49)		
Self-report health status				
Very poor	779 (36.13)	1377 (63.87)	1210.827	0.000
Poor	2735 (56.96)	2067 (43.04)		
Fair	3002 (71.61)	1190 (28.39)		
Good	1276 (82.54)	270 (17.46)		
Very good	355 (86.37)	56 (13.63)		
Having regular physical exercises				
No physical exercise	5011 (61.59)	3125 (38.41)	11.765	0.003
Less than regular physical exercises	1503 (61.02)	960 (38.98)		
Regular physical exercises	1633 (65.11)	875 (34.89)		
BMI				
Underweight	442 (48.79)	464 (51.21)	113.589	0.000
Average	4207 (60.77)	2716 (39.23)		
Overweight	2494 (65.86)	1293 (34.14)		
Obese	1004 (67.34)	487 (32.66)		

CESD: Center for Epidemiologic Studies- depressive symptoms scale

*BMI* body mass index, BMI Categories include Underweight (–18.5 kg/m^2^)/Normal weight (18.5–24 kg/m^2^)/Overweight (24–28 kg/m^2^)/Obesity (28– kg/m^2^)

Hukou: it is an important part of household registration in china and is strictly enforced.Non-agricultural hukou people primarily live in the city and work in factories, public institutions and other workplaces. They have no farm land but have the convenience of city life. Agricultural hukou people primarily live in villages and depend on agriculture and farming for their livelihood

### Bivariate analyses

Based on the CES-D assessment (≥10), the prevalence of depressive symptoms was 37.84% in the total population, 30.70% in males and 44.30% in females. The prevalence of depressive symptoms was significantly different between males and females.

A low prevalence of depressive symptoms was observed in the 45-49 year-old age group compared with 60-64, 65-69 and ≥70 year-old age group respectively (post-adjustment *P* < 0.003). An even lower prevalence was observed in Male, the married groups, the no-agricultural hukou group, the urban group, the taking activities group, the he group who never experienced a major accidental injury (*P* < 0.05).A high prevalence of depressive symptoms was observed in the illiterate educational level group compared with less than elementary school educational level group, high school educational level group, and above vocational school educational level group respectively(post-adjustment *P* < 0.008). An lower prevalence was observed in the no smoke group compared with current smoke group and former smoke group (post-adjustment *P* < 0.017). Compared with no drinking group, the less than once a month drinking group and more than once a month drinking group have a lower prevalence (post-adjustment *P* < 0.017). The group eating ≤2 meals per day has a high prevalence of depressive symptoms than the group eating 3 meals per day and ≥4 meals per day (post-adjustment *P* < 0.017). The very poor self-report health status group has a higher prevalence of depressive symptoms than other health status groups(post-adjustment *P* < 0.005). The regular physical exercises group has a lower prevalence of depressive symptoms than Less than regular physical exercises group and no physical exercise group (post-adjustment *P* < 0.017). Compared with the average group, the underweight group has a higher prevalence of depressive symptoms(post-adjustment *P* < 0.008), whereas overweight group and obese group have higher prevalence of depressive symptoms (post-adjustment *P* < 0.008) (Table 4).

### Multivariate analyses

To examine the association between depressive symptoms and body weight, we estimated depressive symptoms equation using binary logistic regression. The crude ORs and the associated 95% confident intervals (CIs) are shown in Table 5. We controlled for the socio-demographic characteristics. The estimation results of the depressive symptoms equation are reported in Table 6. The ORs and the associated 95% CIs are reported for males, females and the middle-aged and elderly.

**Table 5:** Estimated crude ORs of depressive symptoms

Variables	Male			Female			Total		
	OR	95% CI	P	OR	95% CI	P	OR	95% CI	*P*
Normal weight	1.00			1.00			1.00		
Under-weight	1.65	(1.35,2.02)	0.000	1.53	(1.26,1.86)	0.000	1.63	(1.42,1.87)	0.000
Over-weight	0.67	(0.59,0.77)	0.000	0.79	(0.71,0.88)	0.000	0.80	(0.74,0.87)	0.000
Obese	0.64	(0.51,0.79)	0.000	0.68	(0.59,0.79)	0.000	0.75	(0.67,0.85)	0.000

*BMI* body mass index, BMI Categories include Underweight (–18.5 kg/m^2^)/Normal weight (18.5–24 kg/m^2^)/Overweight (24–28 kg/m^2^)/Obesity (28– kg/m^2^)

**Table 6:** Adjusting ORs and 95% *CI* for BMI and depressive symptoms

Variables	Male			Female			Total		
	OR	95% CI	P	OR	95% CI	*P*	OR	95% CI	*P*
Age (years)	1.00	(0.99,1.00)	0.212	0.99	(0.99,1.00)	0.069	0.99	(0.99,1.00)	0.000
Education									
Illiterate	1.00			1.00			1.00		
Less than elementary school	0.43	(0.29,0.64)	0.000	0.62	(0.40,0.94)	0.026	0.42	(0.32,0.56)	0.000
High school	0.63	(0.47,0.84)	0.002	0.48	(0.36,0.65)	0.000	0.49	(0.40,0.59)	0.000
Above vocational school	0.91	(0.76,1.09)	0.320	0.89	(0.79,1.00)	0.045	0.81	(0.73,0.89)	0.000
Marital status									
Single	1.00			1.00			1.00		
Married	0.53	(0.44,0.64)	0.000	0.69	(0.59,0.81)	0.000	0.60	(0.53,0.68)	0.000
Hukou									
Non-agricultural hukou	1.00			1.00			1.00		
Agricultural hukou	1.44	(1.19,1.74)	0.000	1.49	(1.24,1.79)	0.000	1.48	(1.30,1.69)	0.000
Current residence									
Urban	1.00			1.00					
Rural	1.16	(1.01,1.35)	0.042	1.26	(1.11,1.44)	0.001	1.21	(1.09,1.33)	0.000
Smoke									0.000
NO smoke	1.00			1.00			1.00		
Former smoke	1.11	(0.95,1.28)	0.183	0.91	(0.73,1.13)	0.381	0.79	(0.72,0.88)	0.000
Current smoke	0.91	(0.75,1.11)	0.353	0.87	(0.61,1.25)	0.447	0.67	(0.58,0.78)	0.000
Drinking									0.075
NO	1.00			1.00			1.00		
Less than once a month	0.87	(0.76,0.99)	0.036	1.35	(1.09,1.67)	0.006	0.89	(0.80,0.98)	0.023
More than once a month	0.87	(0.70,1.06)	0.169	1.27	(1.00,1.63)	0.051	0.95	(0.82,1.11)	0.536
Average hours for one night	0.80	(0.78,0.83)	0.000	0.80	(0.78,0.82)	0.000	0.80	(0.78,0.82)	0.000
Eating meals									0.000
3 meals per day	1.00			1.00			1.00		
≤ 2 meals per day	1.51	(1.27,1.79)	0.000	1.46	(1.25,1.71)	0.000	1.49	(1.33,1.67)	0.000
≥ 4 meals per day	0.60	(0.36,1.00)	0.051	0.94	(0.61,1.45)	0.790	0.76	(0.55,1.05)	0.091
Taking no activities									
No	1.00			1.00			1.00		
Yes	0.79	(0.70,0.89)	0.000	0.83	(0.75,0.93)	0.001	0.82	(0.76,0.89)	0.000
Ever been in major accidental injury									
NO	1.00			1.00			1.00		
Yes	1.58	(1.33,1.87)	0.000	1.28	(1.04,1.59)	0.020	1.42	(1.24,1.61)	0.000
Diseases(0-14)	1.28	(1.22,1.34)	0.000	1.30	(1.25,1.36)	0.000	1.30	(1.26,1.34)	0.000
Self-report health status									
Very poor health	1.00			1.00			1.00		
Poor	0.19	(0.12,0.29)	0.000	0.15	(0.09,0.23)	0.000	0.17	(0.12,0.23)	0.000
Fair	0.18	(0.14,0.24)	0.000	0.22	(0.18,0.28)	0.000	0.20	(0.17,0.24)	0.000
Good	0.35	(0.29,0.42)	0.000	0.33	(0.28,0.39)	0.000	0.34	(0.30,0.38)	0.000
Very good	0.56	(0.47,0.66)	0.000	0.54	(0.46,0.63)	0.000	0.55	(0.49,0.61)	0.000
Having regular physical exercises									0.046
No physical exercise	1.00			1.00			1.00		
Less than regular physical exercises	0.88	(0.75,1.03)	0.123	0.87	(0.76,1.00)	0.053	0.88	(0.79,0.97)	0.015
Regular physical exercises	0.94	(0.81,1.10)	0.469	1.03	(0.90,1.19)	0.654	1.00	(0.90,1.10)	0.929
BMI									0.000
Normal weight	1.00			1.00			1.00		
Under-weight	1.30	(1.04,1.63)	0.024	1.19	(0.96,1.49)	0.114	1.27	(1.08,1.48)	0.003
Over-weight	0.76	(0.65,0.89)	0.000	0.80	(0.71,0.90)	0.000	0.80	(0.73,0.88)	0.000
Obese	0.64	(0.50,0.82)	0.000	0.65	(0.55,0.77)	0.000	0.66	(0.58,0.76)	0.000

*BMI* body mass index, BMI Categories include Underweight (–18.5 kg/m^2^)/Normal weight (18.5–24 kg/m^2^)/Overweight (24–28 kg/m^2^)/Obesity (28– kg/m^2^)

Hukou: it is an important part of household registration in china and is strictly enforced.Non-agricultural hukou people primarily live in the city and work in factories, public institutions and other workplaces. They have no farm land but have the convenience of city life. Agricultural hukou people primarily live in villages and depend on agriculture and farming for their livelihood

The effects of several socio-demographic characteristics on depressive symptoms were similar between genders. The depressive symptoms level showed an age-related decline in the middle-aged and elderly. Compared to the middle-aged and elderly adults who were illiterate, those with more education were less likely to be depressed. For example, the estimated ORs for adults who had Less than elementary school and high school were 0.43 (95% CI = 0.29-0.64) and 0.63 (95% CI = 0.47-0.84) among the men, respectively. Marriage had an ameliorating effect on depressive symptoms. Compared with those who were never married, divorced, or widowed (the single group), the ORs of marriage were 0.53 (95% CI = 0.44-0.64) for men and 0.69 (95% CI = 0.59-0.81) for women. Our study also showed that respondents who lived in rural areas were more likely to be depressed, with an OR of 1.16 (95% CI = 1.01–1.35) for men. A null effect between smoking and depressive symptoms was found in both men and women. Sleeping longer during the night appeared to reduce the risk of depressive symptoms. The number of meals per day also affected depressive symptoms levels. As expected, compared to the respondents had 2 meals per day or less were more likely to be depressed, with an OR of 1.51 (95% CI = 1.27-1.79) for men and 1.46 (95% CI = 1.25-1.71) for women, whereas respondents who have 4 meals per day had no association with depressive symptoms. Chronic diseases played an important role on the likelihood of depressive symptoms for both men (OR = 1.28, 95% CI = 1.22–1.34) and women (OR = 1.30, 95% CI = 1.25–1.36), with an additional chronic disease increasing the odds of depressive symptoms by 27.54% for men and 30.38% for women. Compared with middle-aged and elderly adults who have very poor health, men and women with a better health status were less likely to be depressed.

The effects of other socio-demographic characteristics on depressive symptoms were different between genders. Compared with middle-aged and elderly adults who were non-agricultural hukou, the agricultural hukou were more likely to be depressed, with an OR of 1.44 (95% CI = 1.19–1.74) for men and 1.49 (95% CI = 1.24–1.79) for women. Compared with middle-aged and elderly adults who did not drink, the men who drank less than once a month were less likely to be depressed (OR = 0.87, 95% CI = 0.76–0.99), whereas the women who drank less than once a month were more likely to be depressed (OR = 1.35, 95% CI = 1.09–1.67). Furthermore, the results indicated that adults who were active had a lower propensity for depressive symptoms, with an OR of 0.79 (95% CI = 0.70–0.89) for men and 0.83 (95% CI = 0.75-0.93) for women. Our findings also indicated that adults who had been in a major accidental injury had a higher propensity for depressive symptoms, the ORs of having a major accidental injury were1.58 (95% CI = 1.33-1.87) for men and 1.28 (95% CI = 1.04-1.59) for women. Compared with no physical exercise, the adults who had physical exercise had no association with depressive symptoms.

After adjusting for age, education, marital status, hukou, current residence, smoking habits, alcohol consumption, average hours of sleep per night, eating habits, activity status, major accidental injury, chronic diseases, self-reported health status, and regular physical exercise, compared with their normal weight counterparts, middle-aged and elderly men who were overweight (OR = 0.76, 95% CI = 0.65-0.89) and obese (OR = 0.64, 95% CI = 0.50-0.82) were less likely to be depressed. Compared with the women of abnormal weight, the overweight women (OR = 0.80, 95% CI = 0.71-0.90) had a significantly lower OR for depressive symptoms, and the same as the obesity women (OR = 0.65, 95% CI = 0.55-0.77). For the middle-aged and elderly of both genders, compared with the people of normal weight, the underweight people were more likely to be depressed (OR = 1.27, 95% CI = 1.08-1.48), whereas the people who were overweight (OR = 0.80, 95% CI = 0.73-0.88) and obese (OR = 0.66, 95% CI = 0.58-0.76) were less likely to be depressed (Table 6).

## Discussion

In this study, we investigated the association between BMI and depressive symptoms among the middle-aged and elderly in China. Based on the Chinese version of the CES-D, the prevalence of depressive symptoms was 30.70% in men and 44.30% in women. Our results demonstrate that being obese is negatively associated with depressive symptoms in both men and women. However, we find a positively association between under-weight and depressive symptoms among male and absence of the significant evidence among female. It is also worth mentioning the negative association observed between BMI and depressive symptoms in the total. The results provide new insights into obesity.

The prevalence of depressive symptoms was higher compared with previous survey results. In the sample collected from the 1999 and 2003 Surveys of Health and Living Status of the Elderly in Taiwan [16], the prevalence of depressive symptoms as measured by the CES-D (above the cutoff of 10) was 27.9% in men and 36.2% in women in 2003. The prevalence of depressive symptoms in the Elderly Health Centers between July 1998 to December 2000, in a study conducted in a population aged 65 or older using the Geriatric depressive symptoms Scale criteria, was 4.9% in men and 7.9% in women [15].The rates estimated were slightly higher than the respective rates of 29.2% and 41.1% reported by Woo J [53].Such discrepancies between our results and the previous studies may be a result of methodological differences in diagnosis and the healthy worker effect. Unsurprisingly, the prevalence of overweight and obesity was 28.89% and 11.38%, respectively, in our study, which is lower than in the United States(41.3 and 24.3%, respectively) [54].

As the multivariate analyses showed, the relationship between BMI and depressive symptoms depended on BMI status. The overweight and obese groups revealed a negative association with depressive symptoms, whereas the underweight group showed a positive association, particularly in males, although similar results were also found in females. The overweight and obese male groups showed ORs of 0.67 and 0.64, respectively, which were substantial without the adjustments. This negative association with depressive symptoms remained the same after the covariates of age, education, marital status, hukou, current residence, smoking habits, alcohol consumption, average hours of sleep per night, eating habits, activity status, major accidental injury, chronic diseases, self-reported health status, and regular physical exercise were controlled. However, for the adjusted ORs, these values changed to 0.76 and 0.64, which were similar to the results obtained with the females.

These study results were similar to those of several Asian studies [13–16] that revealed that overweight men and women had a negative association with depressive symptoms. In several Asian studies [12, 55], depressive symptoms were found to be inversely associated with BMI in men. However, no association was found among women. Conversely, another study only found a negative association in women [17]. According to previous studies regarding the association between BMI and depressive symptoms, most cross-sectional studies conducted in the United States show a positive association between obesity (BMI >30) and depressive symptoms in women. However, most cross-sectional studies conducted outside of the United States did not support such associations. In non-clinical and clinical obese samples, higher levels of weight-based discrimination have been found to be associated with greater body dissatisfaction, more severe eating disturbances, higher levels of general psychological distress, and lower self-esteem [2], while our study found positive side. It is better for relieve the weight-based discrimination.

Our findings are inconsistent with the results of many cross-sectional studies from the United States that support a positive association between obesity and depressive symptoms in women. However, our result is similar to the results of several cross-sectional studies in Asian populations [13–16]. Moreover, most cross-sectional studies in Asian men failed to find such associations [12, 55]. The studies conducted in Asia did not separately identify the group with a BMI >30, creating difficulty in comparing our research with American studies. Our results are special primarily because we separately assessed the association between depressive symptoms and underweight obese (BMI > 30), overweight (25 ≤ BMI < 30), normal (18.5 ≤ BMI < 25), and (BMI < 18.5) groups, which were defined by the Chinese criteria [48]. The Chinese categorization of BMI is lower than western countries, but higher than most Asian countries [56], and the cut-off points is more sensitive to the Chinese population.

Several possible explanations account for our findings. First, environmental factors such as cultural backgrounds and dietary habits may play a partial role in the results. Asian countries such as, China, Japan and Korea share similar dietary habits and cultural backgrounds. In addition, in Chinese society, the association between happiness and obesity is described in the well-known idiom of “happy mind and fat body”. Therefore, people tend to gain weight later in life as they acquire good fortune. Second, we may consider biological mechanisms, such as “the jolly fat hypothesis”, which was first proposed by Crisp and McGuiness [11]. They found that overweight men have a lower risk of depressive symptoms and show reduced depressive symptoms as a result of several possible mechanisms, including the higher consumption of certain nutrients that are helpful in reducing or preventing depressive symptoms [24].

### Strengths and limitations

Our study has a number of strengths. Firstly, the data consisted of a representative and large sample size of a wide-scale, nationwide survey, which included respondents who were 45 years and older. Secondly, multivariate analyses were used to account for the association between depressive symptoms and obesity according to gender, which allowed us to identify gender-specific patterns of association between BMI and depressive symptoms, and we separately assessed whether depressive symptoms are associated with the obese (BMI > 30) groups and overweight (25 ≤ BMI < 30), which were unique defined by Chinese criteria. This analysis based on different body weight groups provided evidence that the association between BMI and depressive symptoms may depend on the severity of obesity in Asia.

However, our study has a number of limitations. Firstly, the cross-sectional nature of the research had a limited capacity to identify a causal relationship between BMI and depressive symptoms. However, any reciprocal relationships have not been previously demonstrated. Secondly, many outcome measures were based on the self-reported questionnaire and subjective report. Although depression was not diagnosed using the CES-D criteria, we used a validated measure and defined depressive symptoms with the cut-off point of 10. Thirdly, there is a possibility of selection bias. To minimize selection bias, the researchers recruited their subjects by multi-stage stratified probability proportionate to size (PPS) sampling. Fourthly, the small sample size could cause statistical insignificance. The proportion of underweight subjects was only 6.89% of the total subjects, which could lead to a broad CI. If our research included more subjects belonging to the underweight group, the association of that group with depressive symptoms may have resulted in more significant results.

## Conclusions

The result recently conducted studies demonstrate a significant inverse relationship between BMI and depressive symptoms in both men and the women, which supports our “fat and jolly” hypothesis. Obesity may protect the middle-aged and elderly against depressive symptoms. Health policy-makers need to make intervention plans to change the weight bias. However, more studies need to further confirm the fat and jolly hypothesis.

## Abbreviations

BMI: Body mass index
CES-D: Center for Epidemiologic Studies- depressive symptoms scale
CHARLS: China Health and Retirement Longitudinal Study
CIs: Confidence intervals
ORs: Odds ratios
PPS: Probability proportionate to size
